# Preventing violence against children in schools (PVACS): protocol for a cluster randomised controlled trial of the EmpaTeach behavioural intervention in Nyarugusu refugee camp

**DOI:** 10.1186/s12889-019-7627-y

**Published:** 2019-10-15

**Authors:** Karen M. Devries, Camilla Fabbri, Elizabeth Allen, Vivien Barongo, Elizabeth Shayo, Giulia Greco, Michael Kaemingk, Mary Qiu, Rachel Steinacher, Wietse Tol, Katherine Rodrigues

**Affiliations:** 1 London School of Hygiene and Tropical Medicine, 15-17 Tavistock Place, London, WC1H 9SH UK; 2 National Institute for Medical Research, 2448 Barack Obama Dr, Dar es Salaam, Tanzania; 3 Behavioural Insights Team, 195 Montague Street, 14th Floor, Suite 1201, Brooklyn, NY 11201 USA; 4 Innovations for Poverty Action, Regent Business Park, 3rd Floor, Wing-B, Plot 172, Chwaku Road, Mikocheni, Dar es Salaam, Tanzania; 5 Johns Hopkins Bloomberg School of Public Health, 624 N. Broadway, Hampton House 863, Baltimore, MD 21205 USA; 6 International Rescue Committee, 122 E 42nd St, New York, NY 10168 USA

**Keywords:** Violence against children, School violence, Refugee, Emergency, Corporal punishment, Emotional violence

## Abstract

**Background:**

We aim to test the effectiveness of the EmpaTeach intervention to prevent physical violence from teachers to students in Nyarugusu Refugee Camp, Tanzania. EmpaTeach is a 10-week, 14-session, classroom management and cognitive-behavioural therapy-based intervention for groups of teachers for delivery by lay personnel in resource-constrained settings.

**Methods:**

We will conduct a two-arm cluster randomized controlled trial (RCT) with parallel assignment and an approximately 1:1 allocation ratio. All primary and secondary schools in Nyarugusu will be invited to participate. Whole schools will be stratified according to whether they are Congolese or Burundian, and primary or secondary schools, then randomised to active intervention or wait-list control conditions via a public meeting with headteachers. We will collect survey data from *n* = 500 teachers and at least *n* = 1500 students before the intervention, soon after, and at least 6 months after the end of the intervention. The primary outcome measure will be students’ self-reports of experience of physical violence from school staff in the past week, measured using a modified version of the International Society for the Prevention of Child Abuse and Neglect Screening Tool-Child Institutional at the first follow-up after the intervention. Secondary outcomes include emotional violence, depressive symptoms and educational test scores. Analysis will be intention to treat, using repeat cross-sectional data from individuals.

**Discussion:**

If successful, the EmpaTeach intervention would represent one of a handful of proven interventions to reduce violence from teachers to students in any setting. IRC provides an immediate platform for scale up of the intervention via its current work in more than 40 conflict-affected countries.

**Trial registration:**

NCT03745573, registered November 19, 2018 at clinicaltrials.gov, https://clinicaltrials.gov/ct2/show/NCT03745573.

## Background

Violence against children and adolescents is a serious human rights, social, and public health issue. Violence experienced prior to age 18 years is associated with increased risk of a host of adverse future outcomes, including depression, suicide attempts, violence victimization and perpetration, and poor educational outcomes [1–4]. There is emerging evidence that exposure to violence during certain sensitive periods in brain development, including in early adolescence [5] carries particular risk, making adolescence a key period for prevention and response to violence.

School is one of the most common settings where those under 18 years may experience physical, sexual and emotional violence[6]; and in some countries, school staff may be one of the most common perpetrators of violence against children [6]. Nationally representative surveys in Kenya and Tanzania indicate that more than 40% of adolescents have experienced violence from an authority figure, most often teachers [7, 8]. Levels of violence may be even higher in emergency situations where school staff and students have been displaced, may have recent histories of trauma, and face ongoing adversities. Each of these can contribute to an increased likelihood of violence. Few statistics document the prevalence of violence against children and adolescents in schools in refugee camps, but a mixed methods study conducted in Nyarugusu Camp in 2016 found that physical and sexual violence were perceived as common and as unacceptable forms of violence by parents and students themselves [9].

Few interventions have been rigorously trialled for their effectiveness in preventing violence from school staff to students in any setting. In non-emergency settings, the Good School Toolkit in Uganda (full randomized controlled trial [RCT] completed), and the Irie Classroom Toolbox in Jamaica (small efficacy trial showing success; full effectiveness RCT currently underway), are two exceptions, which are effective in reducing physical and emotional violence from school staff to students [10, 11]. Both of these interventions are implemented in primary schools. The Good School Toolkit is a whole school intervention, involving school administration, teachers and students as well as surrounding communities, which is implemented over an 18 month period. The intervention aims to change school operational culture, and contains about 60 different activities that schools can choose to implement. The Irie Classroom Toolbox is a 5 session intervention aimed at teachers, with monthly follow-up support and a range of materials, designed to improve classroom management and children’s social-emotional skills, based on the Incredible Years Curriculum from the USA.

Despite these successes, there have been no trials of interventions to either reduce violence in schools in emergency settings, or - as far as we are aware - reduce violence from school staff to older adolescents in any setting.

### Aims

The primary aim of this project is to test the effectiveness of the Empateach intervention to prevent physical violence from teachers to students in Nyarugusu Refugee Camp, Tanzania. Our secondary objectives are to assess the impact of the EmpaTeach intervention on students' depressive symptoms, experience of emotional violence and educational test scores. We also plan to conduct a parallel qualitative study, process and economic evaluations.

## Methods

### Design

We will conduct a two-arm cluster RCT with parallel assignment.

### Study partners

Our collaboration currently involves five partners, convened by the International Rescue Committee (IRC), an international non-governmental organization (NGO) delivering services in conflict-affected settings. Since 2016, IRC has been developing and piloting the EmpaTeach intervention in conjunction with the Behavioural Insights Team (BIT), a team of UK and USA based behavioural scientists. IRC implements the EmpaTeach intervention in this study. Innovations for Poverty Action (IPA) is the quantitative data collection partner for the study and collaborates on a range of projects with IRC. IPA is an international organisation which facilitates data collection for large scale quantitative research projects via its local country offices. IRC has been working with IPA to collect data in relation to its projects in Africa for over 5 years.

In July 2018, the London School of Hygiene and Tropical Medicine (LSHTM) was invited to join the collaboration to conduct an independent evaluation of the EmpaTeach intervention. LSHTM leads the trial and quantitative aspects of the process and economic evaluations; the Tanzania National Institute for Medical Research leads the qualitative aspects of the research.

### Setting

Nyarugusu refugee camp in Kigoma, Tanzania, was established in 1996 to host refugees fleeing conflict in Democratic Republic of Congo and is one of three refugee camps in Kigoma region. According to the latest UNHCR data, the camp hosts around 150,000 refugees, of whom 80,000 are Congolese refugees who have been in the camp since its opening and around 70,000 Burundian nationals who have found home in the camp after the conflict in Burundi broke out in April 2015. Many Burundians were displaced for longer periods previously in Tanzania due to recurring inter-ethnic violence.

The Camp is made up of 146 villages, spread across 14 zones. Zones are largely divided by population origin: zones 1–7 and 14 host Congolese refugees and zones 8–13 host Burundian refugees. The population is very young, with 58% of residents under 18 and only 3% of adults older than 60 years of age. The two most common ethnic groups in 2017 were Hutu (47.8%) and Bembe (42.8%) and most refugees in the camp speak Kiswahili or Kirundi. Refugees are not allowed to engage in formal employment while in Tanzania according to the Refugees Act, and the government enforces restrictions on the movement of refugees once they are hosted in a designated area. Therefore, the Camp population largely relies on food and commodities distribution by NGOs and United Nations agencies active in the camp.

IRC is responsible for all education activities in the camp and for protection and gender-based violence support services. Schools serve Congolese or Burundian students; children in the camp learn the curriculum of their country of origin. The language of instruction is French for the Congolese refugees, while Kirundi is used as the language of instruction until Grade 5 for the Burundian refugees, and after Grade 5, French becomes the medium of instruction. Kiswahili and English, which are the languages in the host country and in the region, are delivered as subjects. According to a recent joint education needs assessment conducted across the three refugee camps in Kigoma region, 21% of Burundian refugee children attend pre-school, 78% are in primary school and only 3% attend secondary school. Among Congolese refugee children, 45% attend pre-school, 98% attend primary school and 60% attend secondary school. The children most likely to be out of school within the refugee community are children from very poor families, orphans, unaccompanied minors, children with disabilities and adolescents and youth who have reached secondary and post-secondary levels [12]. More than 70% of child refugees report feeling safe at school, though a household survey revealed a number of risks associated with commuting to school or being in school including petty thieves and robberies, natural hazards, sexual violence, and violence in schools [12]. Around 30% of households in two of Kigoma’s refugee camps reported incidences of sexual violence and harassment experienced in camps. School safety is undermined by unsanitary bathroom conditions, and dilapidated and poorly maintained buildings. Corporal punishment appeared to be common in classrooms, though not formally reported on [12].

### Participants

At the school level, all 27 primary and secondary schools in Nyarugusu refugee camp in Tanzania will be invited to participate. The intervention is delivered to individual teachers, and all teachers working in intervention schools will be eligible to receive the intervention. The hypothesized intervention effect will be in all students being taught by participating teachers; however, we will measure effects of the intervention in students who are aged 9 years and over as they are better able to respond to survey questions. Data are being collected from both students and teachers.

### Sampling and recruitment

Permission has been granted to approach schools in Nyarugusu camp by IRC, which is responsible for education provision. Schools will be recruited by approaching headteachers, explaining the research and intervention elements of the study, and inviting the headteachers to consent to school participation. For participation in survey research activities, teachers will be randomly selected from lists of all teachers in the schools. A simple random sample of approximately 500 teachers will be invited to participate in an individual survey. At least 1500 students aged 9 years and over in participating schools will be randomly selected from lists of all students aged 9 years and over in the schools. We will oversample by about 10% to allow for children not found, non-response and refusal to participate (so in total we will invite about 60 students per school; *n* = 1500 assuming 25 schools agree to participate). We will conduct cross-sectional surveys at each time point, but will link data for individuals as far as possible. We also intend to link individual survey data to administrative data held by schools on educational test performance and attendance.

### Consent

Headteachers will be approached by the research team for permission to conduct research in their schools and to participate in the intervention. Headteachers will be invited to provide written consent to participation on behalf of individual students aged less than 18 years. Individual students under aged 18 years will be asked to provide assent for participation; students aged 18 years and over will be asked to provide written informed consent. Individual teachers will be asked to provide written informed consent to participate in teacher surveys. During consent procedures it will be communicated to all individual respondents that participation in study activities is completely voluntary and that individuals will be free to withdraw from the survey at any point in time without any penalty. Teachers will be informed that if an interviewer feels that they are putting children at risk of serious violence, a child protection officer may need to be notified. Students under 18 years will be informed that if an interviewer feels their safety is at risk, a child protection officer may need to be notified. Students over 18 years will also be offered referral for further support. This is described further in the child protection section below.

### Survey procedures

**Fig. 1:**
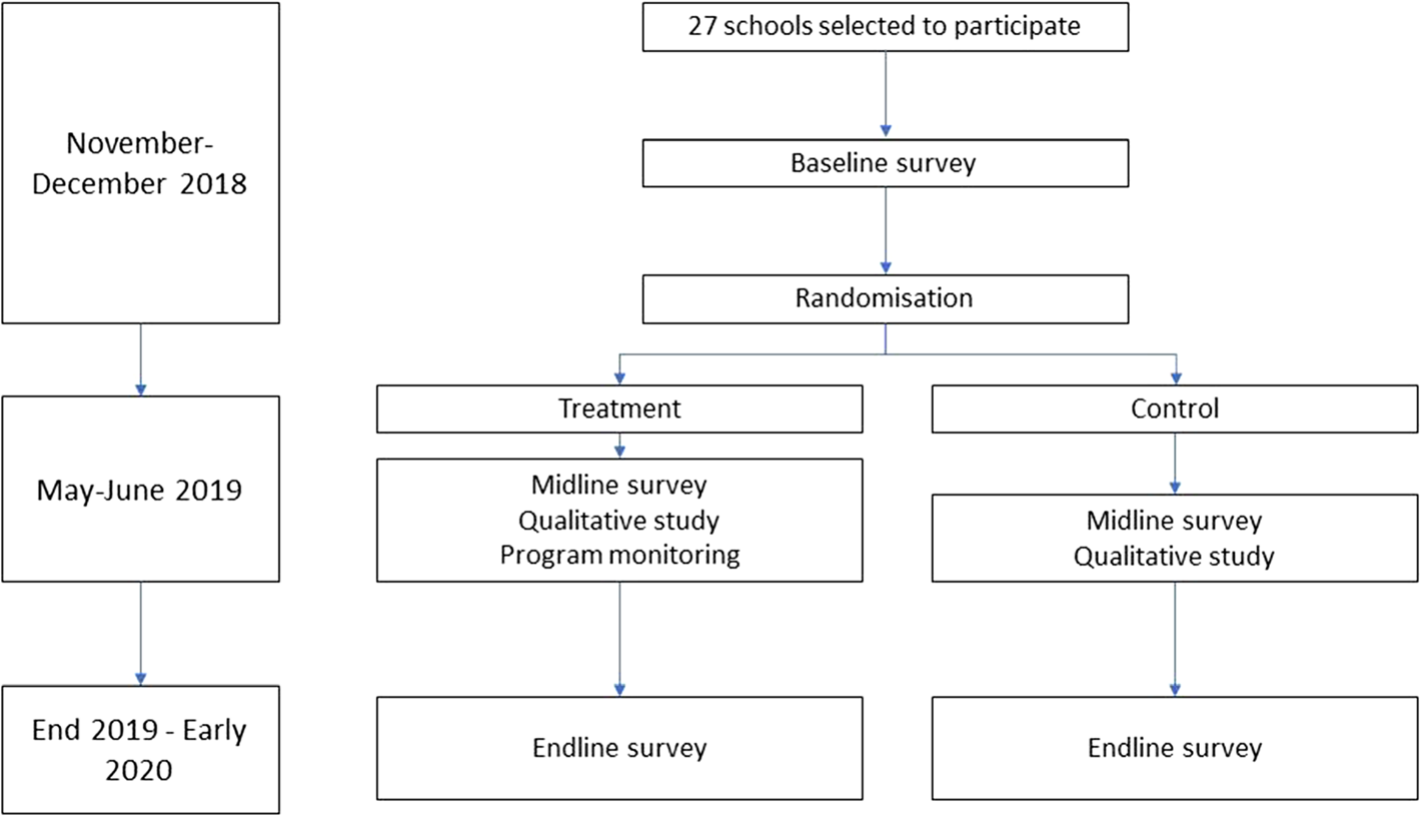
Flow diagram

We will conduct surveys at three time points: a baseline prior to the intervention, a midline soon after the 10-week intervention; and an endline at least 6 months after the end of the intervention (Fig. 1).

Data will be collected at each time point from students and teachers during individual interviews by Kiswahili or Kirundi-speaking interviewers. Before each round of data collection interviewers will receive 2 weeks of in-depth training on how to ask sensitive questions in a non-judgemental manner, survey procedures, and child protection procedures. Individual interviews will be done using a questionnaire programmed into tablet computers. Interviews with students will be conducted at schools, in places where interviewer-participant pairs are out of earshot but within sight of others, to protect confidentiality and ensure child safety. Female interviewers will interview both male and female participants; male interviewers will interview only male participants.

Survey data collected by tablet will be stored on a password protected database that is online-accessible only to senior study personnel, and backed-up daily in encrypted folders. The electronic master database will be held on IPA’s secure server. Devices will be password protected at all times to prevent accidental discovery of personal information by third parties during data collection. Data will contain identifying information as we intend to link data from individuals over time, and to link individual survey data with administrative data held by schools.

### Interventions

The aim of the EmpaTeach intervention is to improve student and teacher well-being, self-regulation, teacher classroom management, and teachers’ use of positive discipline techniques. Teachers in the intervention condition will receive a 10-week group intervention. Groups will meet 14 times for 1–1.5 h length sessions, which will be led by peer teachers. The intervention uses cognitive behavioural therapy techniques to change negative thought and behaviour patterns related to corporal punishment. The teachers will receive information on alternatives to corporal punishment, planning exercises and reinforcement SMS, and because the intervention is in a group setting, social support to change their behaviours. They will discuss their experiences and challenges in group sessions. Table 1 contains a detailed description of the EmpaTeach intervention.

**Table 1:** Description of the EmpaTeach intervention

Aims and Content	
1) To Help teachers to focus on their values, to facilitate receptivity to new information and to improve their self-efficacy and motivate them;	
2) To provide Information about the harmful effects of corporal punishment on children’s health, reflection exercises to build teachers’ empathy for children experiencing corporal punishment;	
3) To provide Information about alternative discipline techniques for classroom management, including de-escalation strategies and techniques to reward positive behaviours;	
4) to provide Information and exercises to improve teachers’ emotional regulation, based on cognitive behavioural therapy, including re-framing and de-escalation; identification of triggers for impulsive reaction in stressful situations and exploring how thoughts and feelings lead to different reactions;	
5) To Help teachers to plan for change, by creating specific actions plans to respond to student’s positive and negative behaviour, and taking account of contextual reasons for student behaviour (for example, students falling asleep because of hunger or illness, or refusing to stand up during menstruation);	
6) To Highlight people’s potential to change and adapt, alerting teachers that children’s behaviour may become worse before it becomes better as children test boundaries and adapt to new classroom methods; building teachers’ confidence in their own ability to adapt; and training teachers to reward and reprimand specific behaviours rather than character traits in children;	
7) To Facilitate a group support system, so teachers can discuss their experiences withothers and receive social support.	
Format	
A BIT program developer, IRC education technical unit staff, and local refugee incentive workers provide a 3-day training to 85 teachers who have been nominated as group coordinators by their peers. Each group coordinator facilitates the 10-week programme with a group of 6–11 teachers. The first 4 sessions are condensed into two four-hour sessions delivered over two weekend days. The remainder of the sessions are held weekly until the end of the programme, and last about 1–1.5 h each – with the exception of Weeks 5 and 11 when groups meet a second time to further engage with the techniques they have learned to date by playing an interactive learning game. The teachers do homework each week, taking about 30 min. They also receive 2 SMS per week from their group coordinators to reinforce aspects of the group sessions or homework. Each session starts with a review of the previous week’s session, reflection on key concepts and sharing of homework, including any challenges encountered. This is followed by an introduction of a short slogan capturing the main learning of the session. This is followed by a series of stories that illustrate a hypothetical but common classroom situation and reflection activities, and presentation and discussion of simple classroom management and self-regulation activities they can use, followed by homework that allows real-world practice of new techniques in teachers’ own classrooms	
Materials.	
A booklet developed specifically to self-guide teachers through each of the 14 sessions in the programme was developed by BIT and IRC in English, and translated into Kiswahili and Kirundi. The booklet contains learning materials for all sessions and space to complete homework assignments. For six of the sessions, there are accompanying videos that were produced locally as part of the intervention. A shared tablet computer is required for each group to view the videos during the sessions. Session 5, which involves learning how to co-create classroom rules with students, requires a large piece of posterboard paper, a marker, and tape or glue to affix the paper to the wall. All teachers are served lunch during the introductory meeting and the programme ending party. Groups decide where they want to meet, and most make use of empty classrooms at schools.	

### Wait-list control

These schools will receive no specific interventions related to violence prevention during the study, but will receive the intervention after the study is over if it is shown to be effective and funding permits.

### Outcomes

The primary outcome will be past-week prevalence of physical violence from teachers to students, measured by students’ self-reports using a modified version of the International Society for the Prevention of Child Abuse and Neglect Screening Tool-Child Institutional (ICAST) [10, 13]. This will be assessed at midline. Secondary outcomes will include: students' self-reports of physical violence from school staff assessed at the endline; students' self-reports of emotional violence from school staff at midline and endline; students' depressive symptoms as measured by the Mood and Feelings Questionnaire [14] at midline and endline, and student attendance and educational test scores on standardised tests, using administrative data from the camp schools, assessed at the end of the school year. The questionnaire based measures have been adapted, cognitively tested and piloted by IRC and BIT in a neighbouring refugee camp with a similar population of teachers and children aged 7 and above, and by IPA in Nyarugusu refugee camp. Based on this process children aged 9 and above were deemed reliably able to answer questions on the above measures.

### Other measures

We will include a number of measures which may mediate or moderate the effects of the intervention, including: teachers’ reports of the use of physical and emotional violence; teachers’ mental health and history of trauma and violence; students’ self-reports of other forms of violence from school staff and other perpetrators; students’ perceptions of school connectedness, students’ perceptions of safety and student mental health. Some measures for students will be administered only to those aged 11 years and over, as some content has been deemed by our study team to be only relevant and appropriate for slightly older children, including all questions on sexual violence and PTSD. We have used all measures with children of the relevant age groups previously in other similar studies (although not previously with refugee children). Table 2 contains a summary of the tools used to measure study outcomes.

**Table 2:** Tools used to measure study outcomes

Construct	Measure
Disability	Washington Group Short set [15]
Attitudes towards school violence^a^	From the Good Schools Study [10]
Students only	
PTSD^a^	Child PTSD Symptoms Scale Self Report [16]
Mental Health	Moods and Feelings questionnaire [17]
Violence from peers	Adapted ICAST from the Good Schools Study [10]
Violence from caregivers	Adapted ICAST from the Good Schools Study [10]
Violence from others	Adapted ICAST from the Good Schools Study [10]
School connectedness	From the Good Schools Study [10]
Engagement with learning	Engagement vs. Disaffection with Learning questionnaire [18]
Teachers only	
Teacher PTSD	Harvard Trauma Questionnaire (part 1, 4) [19]
Teacher IPV	WHO [20]

^a^These will be only administered to children 11 years old and older

### Sample size

Assuming 25 of 27 schools in the camp agree to participate; an intra-class correlation coefficient (ICC) of 0.10 [10]; a significance level of 0.05; 80% power; and a 50% prevalence of past week violence, [10] we would be able to detect a 19% difference in prevalence of physical violence from school staff to students if we surveyed 50 students per school. We thus plan to invite 60 students per school to participate to allow for non-response (n = at least 1500 in total). We also plan to survey about 20 teachers per school (*n* = 500 teachers in total) to explore the intermediate outcomes of the intervention. We planned to stratify randomisation by predictive factors to ensure that the ICC is at the lower end of the range, including whether school are mainly Congolese or Burundian, and whether they are primary or secondary.

### Interim analysis

This is a behavioural intervention which is considered low risk, so no interim data analyses are planned during intervention implementation.

### Allocation

A stratified allocation list will be produced by LSHTM. Allocation will take place at a public meeting of all headteachers, where each headteacher or school representative will place their school name in an opaque bag according to their stratum. Names will then be drawn in turn from each stratum specific bag by one person nominated by the group, and allocated to either the intervention or wait-list control condition in the sequence on the allocation lists.

### Blinding

Due to the nature of the intervention, participants, implementers and research partners should be considered unblinded. The statistician performing the main trial analysis will be blind to allocation.

### Contamination

We have chosen to randomise at the school level to minimise possible contamination. As the intervention is mainly delivered to groups of teachers in a targeted way, we do not expect much natural diffusion due to the nature of the intervention itself. We expect that teacher transfer will be our main possible source of contamination. Based on 2017 data, 10% of teachers transferred to a different school during the school year (although we are informed that these levels are higher than normally expected due to a reorganisation of schools in 2017). We recognise that this contamination potentially reduces the observable effect, and we intend to collect data to assess whether contamination may have taken place.

### Statistical methods

All primary analyses will be carried out according to the principle of intention-to-treat (ITT) and using Generalised Estimating Equations (GEE) to take into account clustering at the school level. Our main analysis of the primary outcomes, and other secondary outcomes will be based on cross-sectional analyses comparing the outcome at midline for two main reasons. One, the intervention will be delivered to all teachers in a given school and is thus expected to impact on all pupils, not just on those pupils who were present at baseline. Two, the literature suggests that in cluster randomised trials, when migration into or out of the clusters is high over time, the baseline cohort may not remain representative of the cluster and therefore repeated cross-sectional analysis is preferred to minimise bias. However, where possible we will link student data across time points and will use analyses that include all students at all time-points, which essentially provides a repeat cross-sectional analysis with a nested longitudinal cohort.

Data will be analysed using multivariate regression models. We will carry out unadjusted analyses and analyses adjusted for any stratification factors and other pre-hypothesised potential confounders as covariates. Formal testing will be restricted to a pre-specified number of the secondary outcomes. A further small number of exploratory subgroup analyses will be specified in advance. These will include subgroups by sex, ethnicity, primary versus secondary school, baseline level of violence and student’s disability.

Other secondary analyses will include teacher outcomes and will be carried out according to the principle of ITT using a similar approach to modelling as described for the student outcomes.

### Qualitative study

The main aim of the qualitative study will be to explore the experience of teachers, students and school administration with the intervention, including what they perceive to be violence; to understand what participants felt ‘worked’ and ‘didn’t work’, and to gain insight into the potential mechanisms of action for teachers and for students. We will conduct semi-structured individual interviews with approximately 10–30 teachers and 10–30 students at three time points at the start of the intervention, during the intervention or immediately after the intervention, and at around 6 months after the intervention. We will attempt to follow the same participants over time. Additionally, we will conduct semi-structured key informant interviews with a sample of approximately 20–40 headteachers, discipline teachers, and group coordinators to understand barriers and facilitators to implementation and contextual information about schools and groups that may have influenced uptake of the intervention. We will conduct approximately 10–12 focus groups with students and teachers. Questions asked will differ based on whether teachers and students are in intervention or control schools. The sample will be weighted so that more participants (approx. 80%) are in intervention schools. Participants will be purposively selected based on analysis of baseline data and recommendations from stakeholders.

Interviews and focus groups will be conducted in Kiswahili, Kirundi or French by a trained research coordinator. These will take place in private locations convenient and safe for both the interviewee/group participant and interviewer/facilitator. With permission, interviews will be recorded, transcribed and translated. Analysis will be thematic, drawing on techniques from Grounded Theory, including constant comparison and searching for deviant cases. This approach will allow us to answer our main questions about how the intervention might work and contextual factors that might affect this, and also for other salient themes to emerge from the data that will provide further information about context more generally.

### Economic evaluation

An economic evaluation will be conducted to estimate the costs and cost-effectiveness of the EmpaTeach intervention. A combination of top-down and ingredients-based costing approaches will be used to generate total cost estimates for each school site. All costing will be estimated from the provider’s perspective and financial and economic costs will be calculated for all inputs. The results of cost analysis will assess the costs of setting up and running the intervention, describe the distribution of costs across different forms of inputs, the unit cost per student reached, the cost of delivering all activities in the intervention site and the cost per unit of measure for selected intermediate intervention outcomes. Outcome measures for the cost-effectiveness analysis will be the change in violence outcomes (cost per case of violence averted). We will estimate the incremental cost effectiveness of the intervention relative to the status quo (represented by the control sites). The cost-effectiveness measure proposed here will be compared to similar violence-prevention programmes in the region and it will inform programme replication, scalability and financial sustainability.

#### Harms and unintended effects; child protection procedures

This is a low risk behavioural intervention; however, it will be delivered to a vulnerable population—adult refugees. Outcomes will be measured primarily in child refugees. Based on our experience doing research on preventing school violence in other populations, these forms of violence are highly normalised among teachers and students, and we do not anticipate that discussion of school violence will itself prompt any adverse reaction. In particular for students, who are not direct recipients of the active intervention, we do not anticipate any adverse effects. It is plausible that there could be unintended harmful consequences for teachers. The intervention involves inducing empathy for students and reflection on use of violence. Given that the teachers are refugees themselves, many of whom will have been fleeing violence and insecurity and may have been victimized in their homes and schools, it might be that some teachers reflect on and revisit their own traumatic experiences which might cause some emotional distress. Based on IRC’s pilot work we think that this is unlikely, however we will monitor teacher emotional distress at the first follow-up survey. We will also monitor whether certain forms of emotional violence, such as yelling or shouting, from teachers to students increase in the intervention group at the first follow-up survey.

During research activities, a subset of children is likely to disclose experience of serious violence or mental health conditions which will necessitate referral for specialist support. We have comprehensive protocols in place to ensure that these children are identified and referred. Children will be referred based on their answers to survey questions. For example, those who disclose recent sexual violence will be referred immediately to child protective services and to the health centre. Prompts will automatically appear on tablet computers at the end of each interview, based on what children have disclosed. These prompts will contain scripted interview finishes and will alert interviewers when they need to refer children for further support. A dedicated child protection officer and protection caseworkers from IRC will be on call at all times during data collection periods to facilitate referrals.

#### Governance

LSHTM is the sponsor for the trial. The study has been approved by the London School of Hygiene and Tropical Medicine (16000–1) and Tanzania National Institute for Medical Research (NIMR/HQ/R.8a/Vol. IX/2920) ethics committees. The trial is overseen by KD and managed by CF. EA is the study statistician. IPA manages data collected during the study, including data related to child protection referrals. IPA staff report to LSHTM staff during the study to ensure independence. IRC initiated the study and are responsible for implementing the intervention and also for child protection responses as described above. The trial is overseen by a trial steering and data monitoring committee; comprised of an independent chair, a statistician, an expert member, the PI, the study manager and study statistician. Representatives from NIMR, IRC, IPA and BIT are invited as observers at the discretion of the chair.

The main data monitoring concerns for this trial relate to ensuring that cases where children have been exposed to severe forms of violence are detected and referred for additional support services. Detecting these children is likely to occur during data collection, rather than during the intervention implementation itself. Comprehensive referral plans have been developed for referral of cases detected during research as described above. At the end of the study, the number of referrals and a summary of actions taken will be presented to the trial steering committee and the LSHTM and NIMR ethics review boards. It is important to note that normally responses to child protection concerns would not be handled by the same NGO implementing the intervention. However, in Nyarugusu Refugee Camp, IRC is responsible for the provision of both education and violence response services.

## Discussion

To our knowledge, this study includes the first randomized controlled trial of an intervention to prevent violence in schools in an emergency setting, and will be one of very few RCTs of an intervention to reduce violence from teachers to students in any setting [10, 21–23]. Violence against children is widespread, with more than one billion children estimated to experience violence each year globally, and teachers are an understudied but important perpetrator of such violence [6]. There is thus a clear need for further trials of interventions to prevent physical and emotional violence from school staff to students.

Prevention in emergency settings may be even more crucial - given that prior exposure to conflict and trauma are associated with increased mental health difficulties, and future use and experience of violence at the individual level [24–26]. Displaced and refugee children are likely to be a key population with whom to intervene to stop cycles of violence, and school provides a good opportunity for doing so. For a displaced child, school may represent a stabilizing, normalizing influence after a period of extreme upheaval and adversity. Young people in schools in emergency contexts may be especially open to new ideas about non-violence, if they are introduced and practiced appropriately.

EmpaTeach is also the first intervention to prevent violence from school staff that we are aware of to draw on techniques from cognitive-behavioural theory, which is a widely used and effective method of behavior change across a range of different fields. The intervention has been designed in collaboration with teachers in a refugee camp, and is thus closely adapted to the local context. It is designed to be implemented by teachers themselves with no specialist mental health qualifications.

### Strengths and limitations

We have chosen a repeat cross-sectional design for our trial, which should minimize risk related to possible attrition stemming from both ongoing resettlement of Burundian refugees and school re-organisations. Burundian refugees in the Camp have the option to voluntarily return home; but as of November 2018 only 6000 of 70,000 living in Nyarugusu Camp had signed up to return. We therefore expect that the number of Burundian students and teachers who are resettled is likely to be small during the period of the study. Obtaining a full sampling frame (in this case, a list of all students and teachers for each Camp school) has also proved challenging, with several re-organisations of schools involving large scale transfers of students which occurred in 2017 and 2018 meaning that school rosters are not up to date. We have also stratified allocation to intervention or control conditions by whether schools are Burundian or Congolese, and thus any outflow of Burundian teachers and students, or transfers between schools, should thus be balanced across arms.

There is also the possibility of another influx of refugees if there is another outbreak of conflict in a surrounding area. If the camp receives another influx of refugees, children would be admitted into existing schools (and/or new schools would be created, depending on the number of new students). Whether additional teachers could feasibly be trained would depend on the timing of the influx, and whether this aligned with the programme implementation period. In this event, we would attempt to document and explore the impact of this via our qualitative and quantitative implementation research.

The intervention is designed for teachers and students, and thus is not designed to directly affect violence outside of schools. In Nyarugusu Camp, about 50% of children are registered with primary or secondary schools, meaning that the intervention will not directly reach about half of the children in the Camp, and our research results should not be interpreted as generalizable to those children who do not attend school in the Camp. It is unknown at present if there are any systematic differences between those children who are and who are not registered in Camp schools.

### Implications

If the EmpaTeach intervention is successful in reducing violence from teachers to students in Nyarugusu Camp, this will represent the first proven intervention model to reduce this form of violence in a conflict setting. IRC currently works in 40 countries, and provides a platform via which to scale EmpaTeach quickly where it already implements education programming.

Empateach and similar interventions are likely to be of interest in non-emergency contexts as well. For example, reports by NGOs indicate that secondary school girls and boys in Tanzania consistently describe high levels of violence and harassment in their schools [27]. Teachers discuss beating students to cause ‘fear’ and to get them into ‘shape’ [27]. If Empateach is successful, further adaptation of non-emergency contexts should be explored.

In Tanzania, IRC is also a leader within the Education Quality Improvement Programme (EQUIP), a consortium which develops and implements best practices to strengthen the quality of education in seven regions across Tanzania, and to ready such interventions for national scale-up. IRC also provides technical support to the Ministry of Education and Vocational Training on teacher professional development, and NIMR advises the Ministry of Health, Community Development, Gender, Elderly and Children. These connections will be leveraged to advocate for uptake.

## Conclusion

Violence erodes the strong foundation that children need for leading healthy and productive lives, and violates the fundamental right of children to a safe childhood. It is therefore vital to address it as a way to prepare and build a future and productive generation. Our trial will provide some of the first evidence on effective strategies to reduce violence from teachers to students in an emergency setting, and is highly scalable. Further research is needed to develop and test new interventions to reduce this common form of violence.

## Data Availability

Not applicable.

## Abbreviations

BIT: Behavioural Insights Team
ICC: intra-class correlation coefficient
IPA: Innovations for Poverty Action
IRC: International Rescue Committee
LSHTM: London School of Hygiene and Tropical Medicine
NGO: nongovernmental organization
NIMR: National Institute for Medical Research
RCT: randomised controlled trial
